# Morphogenic and genetic differences between *Candida albicans* strains are associated with keratomycosis virulence

**Published:** 2009-07-30

**Authors:** Xia Hua, Xiaoyong Yuan, Bradley M. Mitchell, Michael C. Lorenz, Denis M. O’Day, Kirk R. Wilhelmus

**Affiliations:** 1 Department of Ophthalmology, Baylor College of Medicine, Houston, TX; 2 Department of Microbiology and Molecular Genetics, The University of Texas Health Science Center at Houston, Houston, TX; 3 Department of Ophthalmology and Visual Sciences, Vanderbilt University School of Medicine, Nashville, TN

## Abstract

**Purpose:**

To correlate the morphogenic and molecular traits that affect fungal virulence in human corneas.

**Methods:**

*C. albicans* wild-type strains SC5314 and VE175 were compared using in vitro growth kinetics, filamentation assays, and microarray analysis. Corneal virulence was assessed ex vivo by inoculating *C. albicans* onto superficially scarified human corneas that were processed after 1 and 3 days to measure hyphal penetration. For comparison, DSY459, a *C. albicans* homozygous deletion mutant deficient in secreted aspartyl proteinases (SAP) 4, 5, and 6, was evaluated.

**Results:**

*C. albicans* strain SC5314 was highly filamentous in vitro and more invasive in human corneal explants while VE175 demonstrated limited filamentation and less corneal invasion. Among 6,655 *C. albicans* genes, 9.0% significantly (p<.05) differed by 2 fold or more between SC5314 and VE175. Genes involved in fungal filamentation that were upregulated in strain SC5314 compared to VE175 included *SAP5*, *SAP6*, and other hypha-associated genes. Compared to wild-type strains, DSY459 had intermediate filamentation and stromal penetration.

**Conclusions:**

Fungal genes involved in filamentation likely contribute to virulence differences between wild-type strains of *C. albicans*. The corneal pathogenicity of *C. albicans* involves the morphogenic transformation of yeasts into hyphae.

## Introduction

*Candida albicans* is a commensal fungus of the ocular surface [1], an occasional contaminant of ophthalmic devices [2-4], and an opportunistic pathogen of the compromised cornea [5]. In recent clinical series, *Candida* species were isolated from 1% to 5% of eyes with microbial keratitis [6-11].

Improved prevention and therapy of *C. albicans* infections require better understanding of fungal metabolism and growth. O’Day and associates [12] first suggested using *C. albicans* strains having divergent ocular pathogenicity to help identify the mechanisms of fungal virulence for the eye. SC5314, a pathogenic *C. albicans* strain widely used for experimental and genomic studies, rapidly penetrated into the cornea after topical inoculation in immunocompetent rabbits [12,13]. In contrast, another human isolate of *C. albicans*, strain VE175, was significantly less virulent and formed pseudohyphae that were largely limited to the superficial stroma [12]. O’Day [12] hypothesized that “fungal genes that control morphogenesis may be involved”.

Using these same fungal strains in an immunocompetent mouse model of experimental fungal keratitis, Mitchell and associates [14] confirmed that strain SC5314 produced significantly worse keratomycosis than VE175 [15]. Additional studies with fungal mutants provided evidence that fungal filamentation is an important virulence factor for keratomycosis [15-17]. Fungal genes associated with hyphal morphogenesis may account for differences in corneal disease severity produced by disparate strains of *C. albicans* [18].

Our present study further evaluated these two wild-type strains of *C. albicans*. We examined their phenotypic growth characteristics under various environmental conditions and used comparative genomics to explore genetic differences between the two strains. We then developed an ex vivo model of human corneal infection to test the ability of each fungal strain to invade explanted tissue. Finally, we used a *C. albicans* mutant to demonstrate that fungal genes associated with hyphal morphogenesis are important to the pathogenesis of fungal corneal infection.

## Methods

### Fungal strains

*C. albicans* strains included SC5314, a human clinical isolate recovered from a patient with generalized candidiasis [19,20], and VE175, a human corneal isolate obtained from a patient with candidal keratitis [21]. After reviewing the genomic differences between SC5314 and VE175, a mutant strain DSY459 deficient in secreted aspartyl proteinase (SAP) *SAP4*, *SAP5*, and *SAP6* genes (*Δsap4-6*) was obtained from Sanglard and associates who derived this triple-null homozygote from SC5314 using a Ura-blaster cassette [22].

### In vitro growth kinetics

Yeast strains were grown in 1% yeast-extract, 2% peptone, and 2% dextrose (YPD) liquid medium at 30 °C, harvested during exponential growth, and suspended in sterile phosphate-buffered saline (PBS). Optical density (OD) was measured with an Ultraspec 2000 spectrophotometer (Pharmacia Biotech, Princeton, NJ) at a wavelength of 600 nm (OD_600_). A conversion factor of one OD_600_ unit equivalent to 3×10^7^ colony-forming units (CFU)/ml was used to determine fungal concentration [23]. Triplicate samples of 3×10^5^ CFU for each strain were inoculated into 25 ml M199 liquid media (Invitrogen, Grand Island, NY) at pH 6.0, pH 7.3, and pH 8.0 and incubated at 27 °C with continuous shaking. *C. albicans* concentrations were determined spectrophotometrically at 1.5, 3, 4.5, 6, 9, 12, 15, 21, 27, 35, and 48 h post inoculation (pi).

### Filamentation assay

*C. albicans* strains were grown overnight on Sabouraud dextrose agar (Difco, Detroit, MI) for 3 days at 25 ºC. Yeasts were harvested, diluted in sterile PBS to yield 1 CFU/μl, inoculated onto M199 agar plates containing Earle’s salts and glutamine but lacking sodium bicarbonate that were buffered with 2 M Tris-HCl to yield media pH values of 7.3 and 8.0, and incubated at 37 ºC. Colonies were observed daily by inverted microscopy for 10 days.

### Genomic microarray

*C. albicans* strains SC5314 and VE175 were separately inoculated into 10 ml of YPD media and incubated in a room-temperature (22 ºC) shaker until the late exponential growth phase. Ten replicate 1 ml fungal suspensions in 50 ml M199 media at pH 8.0 were held at 37 °C in a 180 rpm incubator, and fungi were harvested after 4 h [24]. Hot acidic phenol extraction was performed to prepare yeast ribonucleic acid (RNA) [25]. The RNA concentration was measured with a ND-1000 spectrophotometer (NanoDrop Technologies, Wilmington, DE), followed by gel electrophoresis to confirm purity and integrity.

Twenty µg of total RNA were labeled by an aminoallyl-derivative protocol using the Micromax RNA labeling kit (PerkinElmer, Waltham, MA). Samples were mixed and hybridized to a glass-slide microarray in the *C. albicans* Array Ready Oligo Set (Operon Biotechnology, Huntsville, AL) composed of 8,117 70-mer oligonucleotides representing 6,655 genes from Assembly 19 of the *C. albicans* genome [26], plus specific and non-specific controls, resulting in slightly more than the predicted 6,354 protein-coding genes due to the inclusion of some *tRNA* genes and spurious genes predicted in earlier assemblies [27]. The array was printed in duplicate on Codelink II (GE Healthcare, Princeton, NJ) slides (Microarrays, Nashville, TN). Labeled samples were mixed and hybridized to the arrays. Two technical replicates and two biological replicates were processed for each sample for a total of eight spots for each gene. Arrays were scanned using an Axon 4000B scanner (Molecular Devices, Sunnywale, CA), and data were analyzed using GenePix Pro 6.0 software (Molecular Devices, Downingtown, PA) as previously described [28]. Inconsistent or saturated spots were filtered, and spot values were calculated by subtracting the background intensity from the median pixel intensity of each spot. Values were then normalized to equalize median intensities between the two channels across the entire array. From the eight replicate spots, geometric means of the ratios and p-values were estimated. Genes having an expression ratio of at least 2 fold difference and p <0.05 between the two strains were grouped into functional categories using information curated by the *Candida* Genome Database [29].

### Reverse transcription (RT) of RNA and RT-polymerase chain reaction (RT-PCR)

Total RNA was separately isolated from *C. albicans* strains SC5314 and VE175 following cultivation for 4 h at pH 7.3 and pH 8.0, respectively. First-strand cDNA was synthesized from 1 μg total RNA with Ready-To-Go You-Prime First-Strand Beads (GE Healthcare) and random hexamers (Applied Biosystems, Foster City, CA). RT-PCR was performed using PuReTaq Ready-To-Go PCR beads (GE Healthcare). Primers for *SAP4* (P1: GTC AAC GCT GGT GTC CTC TT; P2: GCA GGA ACG GAA ATC TTG AG, 197 bp)*, SAP5* (P2: CCG AAT TCC TTT TCC AAA CA; P2: TGG AGC CAT GGA GAT TTT CT, 158 bp)*,* and *SAP6* (P1: GTC AAC GCT GGT GTC CTC TT; P2: TTC ACG AAC ACG AAT TTC ACA; 270 bp) were synthesized (Sigma, St. Louis, MO), with *ACT1* (TGC TGA ACG TAT GCA AAA GG; P2: TGA ACA ATG GAT GGA CCA GA; 186 bp) as the housekeeping gene. Semiquantitative RT-PCR was established by terminating reactions at intervals of 18, 20, 22, 24, 26, 28, 30, 32, 34, 36, and 40 cycles for each primer pair to ensure that PCR products were within the linear portion of the amplification curve. All products were separated by 2% agarose gel electrophoresis and visualized with 0.5 mg/ml ethidium bromide. Fidelity of RT-PCR products was confirmed by comparing cDNA bands and by sequencing PCR products.

### Ex vivo human cornea model

Thirty-six human corneas were obtained from the Lions Eye Bank of Texas, Houston, TX after informed consent for research use was obtained from the decedent donors’ next-of-kin. Donor corneas were maintained at 4 ºC in Optisol-GS (Bausch & Lomb, Irvine, CA) before being transferred to modified supplemented hormonal epithelial medium (SHEM), consisting of equal volumes of Dulbecco’s modified Eagle’s medium and Ham’s F12 medium that contained 5 ng/ml epidermal growth factor, 5 μg/ml insulin, 5 μg/ml transferrin, 5 μg/ml sodium selenite, 0.5 μg/ml hydrocortisone, 30 ng/ml cholera toxin A, 0.5% dimethylsulfoxide, 50 μg/ml gentamicin, and 5% fetal bovine serum, and was buffered with 2 M Tris-HCL to pH 7.3 or 8.0. Corneas were superficially scarified using a 22 gauge needle to produce a 15x15 cross-hatch pattern, similar to a protocol previously described for an experimental fungal keratitis model [14]. A Horizon artificial anterior chamber (Refractive Technologies, Cleveland, OH) fixated the corneal button during superficial scarification, followed by topical application of 10 μl of 1×10^5^ CFU *C. albicans* per cornea. Inoculated corneas were put epithelial side up into a 6 well culture dish (Corning, Corning, NY) so that the sclera was immersed in modified SHEM and the central cornea vaulted upward. Tissues were incubated at 34 °C in 5% CO_2_ with 95% humidity, and culture medium was changed daily. After 24 and 72 h, corneas were embedded in OCT compound (Sakura Finetec, Torrance, CA) and frozen at -80 °C for subsequent histopathologic processing.

### Hyphal penetration

Frozen sections (10 µm) were cut, and three sections were examined for each cornea at 10 μm intervals for 100 µm from the corneal mid-point. Sections were stained with periodic acid-Schiff (PAS) reagent (Sigma-Aldrich, St. Louis, MO). Images were captured of an entire limbus-to-limbus section from each cornea with a DS-Fil digital camera (Nikon, Tokyo, Japan) attached to a Nikon Y-FL microscope. We measured the overall hyphal depth with 10X magnification at 5 different points at equidistant intervals. The entire corneal thickness was then estimated at 5X magnification at the same positions for each section using the NIS-Element 3.0 image analysis system (Nikon). The overall average depth of penetration was calculated as the percentage of corneal thickness. The maximal percentage of hyphal penetration was also estimated from measurements taken at regions of each corneal section demonstrating the greatest depth of corneal penetration. To assure that points of maximum hyphal penetration were selected, results from the three largest hyphal-depth percentages were averaged from 5 measurements taken for each histological section and calculated as percentage of corneal thickness. Results were compared by the Student *t*-test.

## Results

### In vitro comparison of *C. albicans* strains

Strains SC5314 and VE175 demonstrated similar lag, log-growth, and plateau phases. The doubling time of both *C. albicans* strains use the same at pH 6.0 (1.94±0.04 h for SC5314 and 1.92±0.04 h for VE175) and similar at pH 7.3 (2.21±0.04 h for SC5314 and 1.94±0.01 h for VE175) but showed that SC5314 grew slightly faster (2.27±0.03 h) than VE175 (2.72±0.05 h) at alkaline pH (p=0.02). Mock-inoculated control cultures remained negative for growth throughout 24 h of observation. On M199 agar, numerous filaments surrounded SC5314 colonies 24 h after inoculation and continued growing during the following 10 days. Few filaments emerged from VE175 colonies, and while dissimilarity was present at physiologic pH, the difference with in vitro filamentation was more apparent at pH 8.0.

### Genomic comparison of *C. albicans* strains

**Figure 1:**
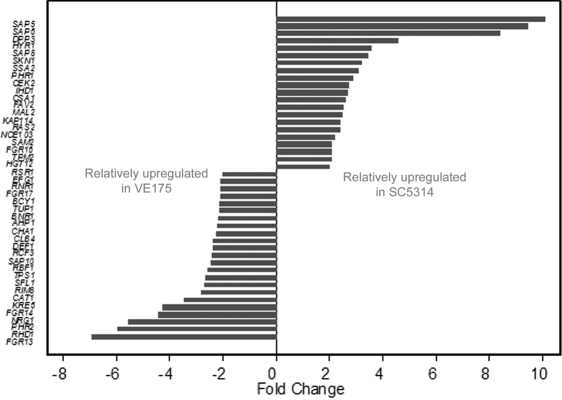
Differential gene regulation comparing strain SC5314 to strain VE175 of 44 *C. albicans* genes involved in fungal filamentation.

**Figure 2:**
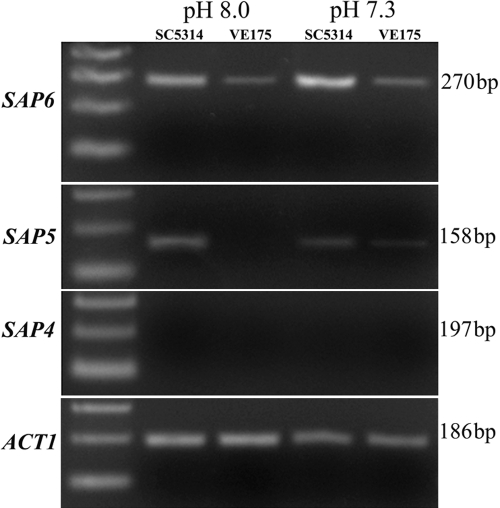
RT-PCR analysis of *SAP4, SAP5,* and *SAP6* expression in *C. albicans* strains SC5314 and VE175 cultured on pH 7.3 and pH 8.0 media. *SAP5* and *SAP6* bands were more apparent from SC5314 than VE175. *ACT1* gene expression appeared similar between both strains at each pH. *SAP4* was not detected.

Among 6,655 genes detected by microarray, 601 (9.03%) genes in SC5314 were significantly (p<0.05) and differently (≥2 fold change) regulated compared to VE175. Of these, 69 named upregulated genes and 116 named downregulated genes were categorized into functional groups (Table 1), including 44 genes involved in hyphal filamentation and virulence (Figure 1). mRNA expression of *SAP4*, *SAP5*, and *SAP6* was further examined by RT-PCR (Figure 2). No difference of *ACT1* expression occurred between SC5314 and VE175 at either pH 7.3 or pH 8.0. mRNA expression of *SAP4* was not detected in either strain at either pH. *SAP5* and *SAP6* mRNA were expressed higher in SC5314 than VE175 at both pH conditions.

**Table 1:** Functional Categories of Genes Expressed in *C. albicans* strain SC5314 compared with VE175 at pH 8.0.

**Functional categories**	**Upregulated genes**	**Downregulated genes**
Filamentation/virulence	*SAP5*, *SAP6*, *DPP3*, *HYR1*, *SAP8*, *SKN1*, *SSA2*, *PHR1*, *CEK2*, *IHD1*, *CSA1*, *FAV2*, *MAL2*, *KAP114*, *RAS2*, *NCE103*, *SAM2*, *FGR16*, *TPM2*, *HGT12*, *RSR1*	*FGR13*, *RHD1*, *PHR2*, *NRG1*, *FGR14*, *KRE6*, *CAT1*, *RIM8*, *SFL1*, *TPS1*, RBF1, SAP10, *RCF3*, *DEF1*, *CLB4*, *CHA1*, *AHP1*, *BNR1*, *TUP1*, *BCY1*, *FGR17*, *RNR1*, *EFG1*
GPI-anchored protein	*PGA26*, *PGA31*, *PGA7*, *PGA34*, *ALS1*, *ECM331*, *PGA45*	*ALS4*, *RBR1*, *YWP1*, *ALS2*, *PGA1*, *PGA57*, *PGA18*, *PGA38*, *PGA37*, *PGA5*
Other cell surface	*HYR2*, *EXG2*	*FMP45*, *MNN1*, *GCA1*, *PIR1*, *ENG1*, *RBE1*, *ALS7*, *ECM17*
Iron metabolism	*FRP1*, *SIT1*, *IRO1*, *HMX1*, *FET35*, *ATX1*, *HAP3*, *FRE9*	*FET3*, *FRE7*
Other metabolism	*PHO89*, *ERP1*, *FRP2*, *PDC12*, *SFC1*, *CA1*, *HAL21*, *IFD6*, *CTN1*, *FAA21*, *AOX2*, *ACB1*	*LEU4*, *GAD1*, *LIP4*, *ACO2*, *CAR2*, PUT1, *HBR2*, *ALD6*, *AAT21*, *FDH1*, *THR4*, *MET3*, *ARO3*, *GLT1*, *BAT21*, *CAN1*, *GIT1*, *IFG3*, *PHO81*, *HOM6*, DLD1, OYE2, DPP1, *OPT9*, *APE3*
Transcription & gene regulation	*ARG1*, *INO2*	*CRZ2*, *CAT8*, *LAP3*, *GST3*, *RME1*, *UPC2*, *ADAEC*, *HHO1*
Membrane transport	*HGT20*, *ENA2*, *FTH1*, *AAP1*, *GIT2*	*HGT17*, *PHO87*, *AQY1*, *IFC3*, *HXT5*, *HGT19*, *CRP1*, *IRT2*, *PHO84*, *CDR4*, *XUT1*, *GIT4*
Others	*XOG1*, *PRN3*, *CAG1*, *TOM22*, *SOD5*, *GRP2*, *MEC3*, *STE2*, *SOL1*, *ARG3*, *RAD16*, *IFM1*	*SSY5*, *SMC3*, *ASM3*, *ECM42*, *TIF11*, *ILV3*, *TPS3*, *LYS22*, *CWH8*, *SYS1*, *CSH1*, *SMC1*, *CCP1*, *ZRT2*, *HSP30*, *CYS3*, *MXR1*, *GLN4*, *SSP96*, *MSH6*, *SWE1*, *POL30*, *SFI1*, *POL1*, *EST3*, *ASR1*, *CDC34*, *AMO2*

Genes in each categorical group were expressed by 2 fold or greater (p <0.05) and are ordered from greatest to least expression ratio. Genes assignable to more than one category are listed only once. In the table, GPI indicates glycosylphosphatidylinositol.

### Corneal virulence

**Figure 3:**
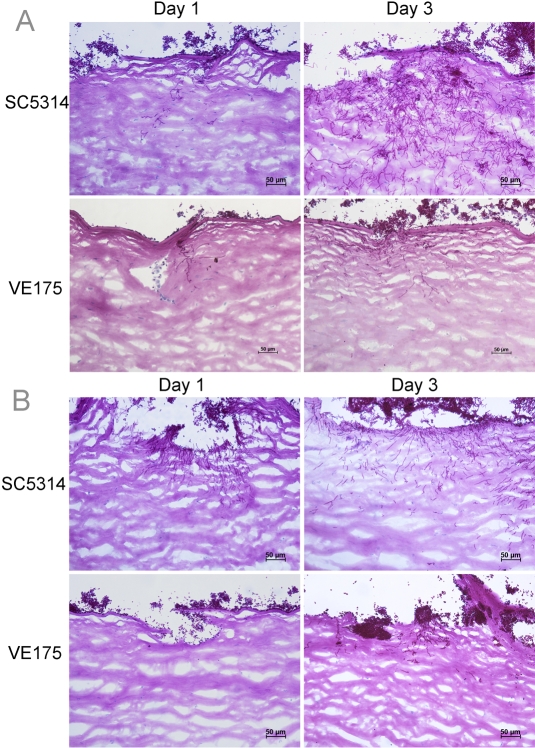
Histopathology of human keratomycosis induced by *C. albicans* strains SC5314 and VE175. **A**: At pH 7.3 SC5314 established superficial infection by day 1 post inoculation, and hyphae proliferated and invaded centrally by day 3. Strain VE175 produced few pesudohyphae in the superficial cornea that did not penetrate through anterior lamellae by day 3. **B**: At pH 8.0 strain SC5314 formed hyphae that extended around superficial scarification site on day 1 post inoculation and continued to invade by day 3. Strain VE175 produced few filamentous forms on day 1 or day 3. Periodic acid-Schiff stain. Scale bars, 50µm.

One day after inoculation of strains SC5314 and VE175 onto human corneas incubated at pH 7.3, fungal hyphae were present in the anterior corneal stroma. By the third day SC5314 invaded toward the central stroma (Figure 3A). VE175, however, failed to continue hyphal invasion between 1 and 3 days pi and remained limited to the anterior cornea. At pH 8.0, hyphal penetration into corneal tissue was consistently greater for SC5314 than VE175. The overall and maximal penetration percentage are shown in Table 2 and Table 3. Initially, few conidia of VE175 were present on the corneal surface and no hyphal formation was observed (Figure 3B). Pseudohyphae subsequently formed with VE175 but did not penetrate to the same extent as SC5314.

**Table 2:** Overall penetration (%) of *C. albicans* strains into explanted human corneas under physiologic and alkaline pH conditions.

**Time**	**pH 7.3**			**pH 8.0**		
	**SC5314**	**VE175**	**p value**	**SC5314**	**VE175**	**p value**
Day 1	8.1±4.9	2.0±2.4	0.12	1.7±0.8	0	0.02
Day 3	19.9±4.6	6.9±7.2	0.06	10.8±4.7	7.6±8.6	0.60

**Table 3:** Maximal penetration (%) of *C. albicans* strains into explanted human corneas under physiologic and alkaline pH conditions.

**Time**	**pH 7.3**			**pH 8.0**		
	**SC5314**	**VE175**	**p value**	**SC5314**	**VE175**	**p value**
Day 1	21.8±5.6	16.0±7.6	0.11	17.0±6.0	0	<0.0001
Day 3	41.9±6.1	16.0±6.7	0.0007	33.7±12.3	18.9±12.5	0.02

### Δsap4-6 deletion mutant

**Figure 4:**
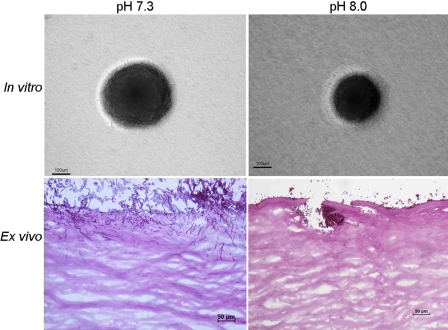
In vitro and ex vivo filamentation of *C. albicans* *∆sap4-6* mutant strain. Compared with SC5314 and VE175, this mutant strain exhibited low but intermediate capabilities to produce filaments around fungal colonies. At day 1 postinoculation, the mutant strain formed hyphae in the superficial stroma at pH 7.3 but did not produce filamentous forms at pH 8.0.

Fungal mutant DSY459 grew well at pH 6.0 and pH 7.3 and slightly more slowly at pH 8.0. Moderate filamentation was found in vitro at pH 7.3 and pH 8.0 (Figure 4). In explanted corneas at 1 day pi (Figure 4), the overall penetration was 5.2%±4.7% at pH 7.3 and 1.0%±2.1% at pH 8.0 (p=0.24), and the maximal penetration was 5.6%±8.5% at pH 7.3 and 2.7% ± 4.1% at pH 8.0 (p=0.38). At day 3 pi, the overall penetration was 11.1%±3.0% at pH 7.3 and 7.2%±2.1% at pH 8.0 (p=0.14), and maximal penetration was 23.8%±5.1% at pH 7.3 and 16.0%±11.3% at pH 8.0 (p=0.049). Compared to SC5314, the maximal penetration of DSY459 was significantly less at pH 7.3 at day 1 (p<0.001) and at day 3 (p<0.001) and was significantly less at pH 8.0 at day 1 (p<0.001) and at day 3 (p=0.003), but there was no difference at the overall hyphal penetration between these two strains at both pH conditions at day 1 and day 3.

## Discussion

*C. albicans* is a common symbiont of mammalian microflora and an important infectious agent. Impaired immunity and altered defenses predispose a host to *C. albicans* ocular infection [5,14], but fungal virulence traits are also involved [16]. Pathogenicity involves a dynamic interaction between susceptible hosts and fungal pathogens [30].

The infectious nature of *C. albicans* and other fungi is partly a result of their morphological plasticity. Microbial survival depends on the ability to detect and to respond to local conditions. During tissue invasion *C. albicans* transforms from blastospores into invasive filamentous forms [16]. This transition from commensal yeasts to pseudohyphae and hyphae results from interrelated pathways that respond to local cues [31,32]. Changes that bring about alterations in fungal morphology include a rise from room to body temperature and a shift in pH.

Adaptation to neutral and alkaline environments by fungi uses the Rim101/PacC signal transduction pathway, a mechanism conserved throughout yeasts and moulds [32-34]. This pathway regulates gene expression via transcription factor Rim101/PacC during the pathogenesis of fungal infection and links the ability to respond to environmental pH with virulence and disease [35].

The Rim101/PacC protein, available as a full-length or processed form, is inactive under acidic conditions and is activated at neutral or alkaline pH by proteolytic processing, becoming capable of inducing hyphal formation and filamentation [33,34]. Proteolysis is regulated in part by upstream members of the pathway including Rim8/PalF, Rim13/PalB, Rim20/PalA, Rim21/PalH, and Snf7 proteins [33,36] and downstream genes including *EFG1*, *PHR1*, and *PHR2* [37-39]. Mechanisms governing fungal morphogenesis are closely related to the development of keratomycosis [16].

Fungi differ in virulence, even among strains of the same species. *C. albicans* strains can produce differing levels of infection severity [40,41]. *C. albicans* SC5314, a widely used strain, shows substantially higher virulence for rabbit and mouse eyes than *C. albicans* VE175, a human corneal isolate [12,15]. Our ex vivo model of human corneal infection confirms that SC5314 rapidly forms hyphae that penetrate into the corneal stroma. On the other hand, based on overall penetration and maximal fungal invasion, VE175 is less invasive and is largely limited to epithelial adherence of blastospores with few pseudohyphae in the superficial stroma.

Both SC5314 and VE175 have similar replication kinetics. However, strain SC5314 readily produced filamentary growth in vitro at neutral and alkaline pH while VE175 had limited filamentation. VE175 also demonstrated less hyphal penetration into corneal tissue compared to pH 7.3. We hypothesize that a defect in filamentation regulated through the Rim101/PacC signal transduction pathway might explain the dissimilarity in corneal pathogenicity produced by these *C. albicans* strains.

Phenotypic differences in virulence can be due to genotypic variations among *C. albicans* strains [42,43]. O’Day [12] pointed out that identifying “the intrinsic genetic differences between such strains may help identify factors responsible for fungal virulence”. Since the genome of *C. albicans* has been sequenced [26], we compared the relative genetic expression of SC5314 and VE175 to seek possible reasons for their disparate pathogenicity.

*C. albicans* has numerous genes involved in nutrient uptake, metabolism, cellular structure, and morphogenesis that are needed for growth and survival. We found that a preponderance of *C. albicans* genes to be similarly expressed in SC5314 and VE175 strains, suggesting that relatively few genes may contribute to virulence differences between these clinical isolates. Nearly one fourth of named genes that were differentially expressed were involved in hyphal formation. Because VE175 displayed reduced filamentation in vitro and within the explanted cornea, the formation of fungal hyphae appears to be part of a causal pathway during infection by *C. albicans*.

The ability of *C. albicans* to produce hyphae in vitro correlates with the production of filamentous forms during experimental corneal infection [16]. Mutations in fungal genes involved in the yeast-to-hyphal morphogenesis of *C. albicans* affect the development of *C. albicans* keratitis [15,44]. Since a mild environmental shift activates a pathway leading to fungal filamentation [31,33], we examined the effect of pH in this study.

The expression levels of genes involved in the pH-dependent Rim101 pathway were largely similar between the two strains, but downstream differences were found. *PHR1*, a pH-responsive gene that encodes a cell-wall glycosidase acting on polysaccharide cross-linking during hyphal morphogenesis [38,45], was significantly downregulated in strain VE175. We also noted that VE175 tended to produce round blastospores that were larger than SC5314 when grown in vitro, resembling a *PHR1*-deletion mutant grown in an alkaline medium [38]. In a neutral-to-alkaline milieu *PHR1* is induced following Rim101p activation, and deletion of the *RIM13* gene attenuates corneal virulence of *C. albicans* [15]. In addition, the relative upregulation of the transcriptional repressor *TUP1* in VE175 could contribute to this strain’s attenuated virulence [16]. These findings indicate that the dimorphic transformation from yeasts to hyphae is incriminated in the onset and progression of *C. albicans* keratitis.

Another virulence factor associated with hyphal morphogenesis is the family of hypha-associated hydrolases [46]. Among secreted aspartyl proteinases [47] Sap6p is required for fungal invasion of the parenchyma and of the cornea [44,48]. Downregulation of *SAP5* and *SAP6* in the less virulent strain VE175 compared to the pathogenic strain SC5314 may partly underlie their different abilities to invade. To explore the role of these proteinases, we used a *Δsap4-6* deletion mutant of *C. albicans*. This knockout strain was intermediate between wild-type strains in the ability to penetrate corneal tissue at a physiologic pH. Proteinases encoded by *SAP5* and *SAP6* may facilitate the penetration of *C. albicans* hyphae through the epithelium and extracellular matrix [49,50].

The molecular mechanisms responsible for myotic infections are emerging. This study used phenotypic screening, comparative genomics, and ex vivo corneal infection to gain better insight into the molecular biology of fungal keratitis. By demonstrating genetic variations between fungal strains having different capabilities of producing keratomycosis, this study supports the key roles of fungal filamentation and hypha-associated proteins in the pathogenesis of *C. albicans* keratitis.
